# Multiple Antibiotic Resistance-Coliform Bacteria in Some Selected Fish Farms of the Central Region of Ghana

**DOI:** 10.1155/2020/6641461

**Published:** 2020-12-04

**Authors:** Cynthia Ayefoumi Adinortey, Denis Worlanyo Aheto, Alex Asomeni Boateng, Rosemary Agbeko

**Affiliations:** ^1^Department of Molecular Biology and Biotechnology, School of Biological Sciences, University of Cape Coast, Cape Coast, Ghana; ^2^Department of Fisheries and Aquatic Sciences, School of Biological Sciences, University of Cape Coast, Cape Coast, Ghana; ^3^Centre for Coastal Management (Africa Centre of Excellence in Coastal Resilience), University of Cape Coast, Cape Coast, Ghana

## Abstract

This study aimed at assessing the quality of water and fishery products as well as the antibiotic resistance status of some selected fish farms of the Central Region of Ghana. Interview guides were administered to farmers to get data on farming practices and antibiotic use. Total coliform loads of catfish (*Clarias gariepinus*), tilapia (*Oreochromis niloticus*), and water samples were determined. Coliforms were identified through various biochemical assays, and their antibiotic resistance patterns were determined. Generally, the total coliform loads of water samples significantly surpassed those of fish samples (*p* < 0.05). The maximum and minimum antibiotic resistance percentages were recorded for ampicillin (96.83%) and gentamicin (6.35%), respectively. Although farmers stated that antibiotics were sparingly used, coliform bacteria isolated exhibited various antibiotic resistance patterns. Four out of six fish farms harboured some coliforms with multiple antibiotic resistance (MAR) indices above 0.2, an indication that these bacteria originated from potentially dangerous sources where antibiotics are regularly used.

## 1. Introduction

As an important sector of the fishery industry involving the farming of aquatic animals such as fish, crustaceans, molluscs, and aquatic plants under controlled conditions, aquaculture plays a crucial role in global fish production [1]. Fish farming also known as pisciculture has, therefore, become an additional source of fish to enhance nutrition and food security in developing countries and to meet the requirement of the increasing global population [2]. The most commonly cultured fish species in Ghana is the Nile tilapia (*Oreochromis niloticus*) representing 80% of total aquaculture production while the remaining 20% comprise catfish (*Clarias gariepinus* and *Heterobranchus* sp.) [3]. Despite the many prospects of pisciculture in Ghana, its management is confronted with some challenges with pollution recognized as the most frequent and devastating problem in aquaculture [4]. Pollution in fish ponds may be of biological, physical, or chemical nature. Infectious agents such as parasites, fungi, and bacteria play a key role in the biological pollution of fish ponds and may, in addition to endangering the well-being of fishes, cause fishery products to be unwholesome. Among infectious bacterial agents, coliform bacteria and *Escherichia coli* have been recognized as important pathogens in fish populations [5]. The microbial assessment of fish and its aquatic environment is critical to provide some vital information about the hygienic status of aquaculture environments. Chemical pollution mainly due to herbicides and pesticides have likewise been documented in fish ponds [4] with very disturbing repercussions on the survival of fish as the latter are vulnerable to such chemicals [6]. Apart from pesticides and herbicides, antimicrobials especially antibiotic residues have been reported in Ghana and elsewhere to be widespread in aquaculture waters, products, and sediments [7–9]. Some studies have linked the presence of antibiotic residues as well as antibiotic-resistant bacteria in the natural environment to several factors, including the continuous usage of antibiotics in farming, animal husbandry, and inappropriate disposal of the various domestic, hospital, and industrial wastes [7, 10–12].

Although antibiotics are very often used in agriculture with the prime aim to reduce economic losses associated with infectious diseases [10], they tend to gradually lose their effectiveness due to their inappropriate usage which subsequently leads to the emergence of antibiotic-resistant bacterial strains. Such resistant bacterial strains can be directly transmitted to humans through the consumption of contaminated food [13] or indirectly through the transfer of their resistance genes to fish pathogenic bacteria or human pathogens, thereby posing a serious global health threat [14]. Unfortunately, because of the high similarity between antibiotics classes that are dispensed in veterinary medicine and those used to treat human infections, these resistant bacterial strains potentially have a serious toll on the treatment of fish and human diseases [15]. It, therefore, becomes essential to probe into the occurrence of a group of bacteria of clinical importance as coliforms in the environment and elucidate their antibiotic resistance patterns.

A few studies have been reported on the use of antibiotics and the detection of multiple antibiotic-resistant (MAR) bacteria in aquaculture settings of the Greater Accra and Ashanti regions of Ghana [7, 16–18]. The present study sought to gather information on management practices adopted in fish farms of the Central Region of Ghana and to investigate the extent of coliform contamination of these fish farms as well as their antibiotic resistance status.

## 2. Materials and Methods

### 2.1. Study Sites

**Figure 1:**
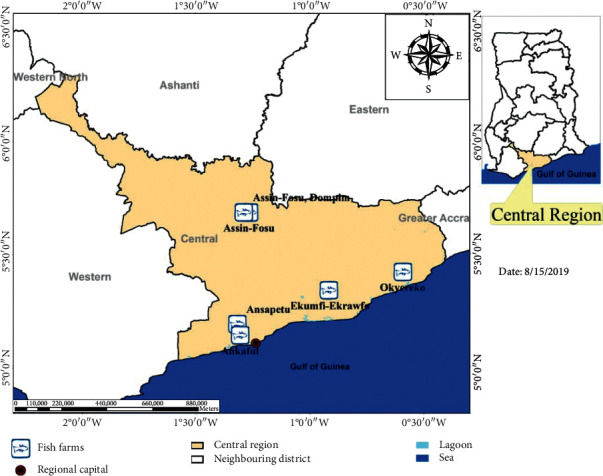
Geographic distribution of fish farms sampled.

The study involved six small-to-medium scale fish farms in active production, purposively selected and located in six towns of the Central Region of Ghana comprising Ansapetu, Ekumfi-Ekrawfo, Okyereko, Ankaful, Assin-Fosu, and Assin-Dompim (Figure 1). With an average stocking density of 5–10 fingerlings/m^2^, the surface areas of the ponds were 6–20 m^2^ and 2.16 m^2^, respectively, for concrete and plastic tanks and 500 m^2^ for the earthen pond. Located in the central part of southern Ghana, the Central Region of Ghana is one of the sixteen administrative regions of the country. The region comprises 20 administrative districts which spread over 9,826 km^2^ thereby representing 6.6% of the total land area of Ghana [19]. By virtue of its various renowned secondary and tertiary cycle institutions and industries as well as famous tourist attraction sites, the region is well known. Except for Cape Coast, the capital of the region, the majority of the districts engage in agriculture, including fishing and the cultivation of cash crops such as cocoa, oil palm, pineapple, and citrus [20]. Though the main occupation in the nine coastal districts is fishing from the ocean, fish farming is practised in the hinterland of the region.

### 2.2. Sample Collection

Sample collection comprising the administration of interview guides as well as the collection of water and cultured species was conducted between 9 : 00 am and 12 : 00 pm on each day of sampling. First-hand information on management practices adopted by farmers, including the use of antibiotics, was obtained through the administration of structured questionnaires after obtaining the farmers' consent. All procedures used on the fish samples were performed according to the animal welfare policy of the Veterinary Directorate of the Ministry of Food and Agriculture in Ghana as well as following the sets of recommendations on animal care and use [21].

Water and cultured fish species were sampled with the assistance of farm managers and by following with slight modifications the guidelines provided by [22]. Water and cultured fish samples were collected from three different spots of each farm: the inflow, the middle, and the outflow parts of the pond. At every sampling spot, approximately 100 mL of water was sampled using sterile bottles at a depth of 50 cm below the water surface while cultured fish species were caught using casting nets or scoops depending on the type of fish-holding facility. Five healthy fishes were randomly taken from each catch and transferred into sterile polyethylene bags. The water and fish samples collected were labelled, put in an ice-chest containing ice blocks, and sent to the laboratory within 3–5 hours where they were kept at 4°C for a maximum of 24 hours after collection before processing and subsequent analyses.

### 2.3. Sample Processing

In order to obtain one representative sample for each farm, all three samples collected from the sampling spots were pooled together based on the sample type [22].

Each of the sets of three water samples collected from every farm was slowly inverted about 10 times in order to get it thoroughly mixed. Subsequently, 3 mL aliquots of each bottle were transferred into a sterile test tube to obtain a 9 mL composite water sample. After thorough mixing, 1 mL of the composite water sample collected from each farm was serially diluted in sterile normal saline solution up the 5^th^ tenfold-dilution factor.

The surface of every fish sample was disinfected using 70% ethanol, and using a sterile scalpel, the fish was aseptically cut through its peritoneal cavity from the pyloric valve to the anus. The intestines were removed, and approximately 0.5 g of the intestinal sample for each sampling spot was transferred into a test tube containing 4.5 mL of sterile normal saline solution. The mixture was vortexed, and 3 mL aliquots of the intestinal sample from all three sampling spots were pooled into an additional sterile test tube to obtain a composite intestinal sample for every cultured fish type and each farm. The resulting 9 mL composite intestinal samples were diluted up the 6^th^ tenfold-dilution factor [22].

### 2.4. Isolation of Coliform Bacteria

Using the spread-plate method, 0.1 mL of each diluted composite water sample, on the one hand, and 0.1 mL of each composite cultured fish sample, on the other hand, were individually pipetted unto the surface of previously prepared sterile MacConkey agar plates.

All inoculated MacConkey agar plates were incubated at 35°C for 16 to 24 hours. Analyses were conducted on each sample in triplicate [22–26].

### 2.5. Determination of Total Coliform Load

After incubation, the colony-forming units (CFUs) of bacteria that developed on the culture plates (ranging from 30 to 300) were counted using a colony counter (Stuart Scientific, UK) and recorded according to samples from which they were recovered. The total coliform load per millilitre (mL) or per gram (*g*) was estimated as the original cell density (OCD) of the sample and determined by using the formula OCD= CFU/DXV, where OCD is the original cell density, CFU is the colony-forming unit counted on the agar plate, D is the dilution of inoculum plated, and V is the volume of inoculum plated [27].

The mean total coliform load of each sample type was determined as the average value of the total coliform load obtained from triplicates.

### 2.6. Storage and Identification of Bacterial Isolates

After counting, an average of 5 morphological different colonies per sample was selected, purified, and subsequently stored on nutrient agar slants in a refrigerator at 4°C where they were kept for subsequent identification and antibiotic susceptibility testing. Identification of stored bacterial isolates was done using biochemical tests, including the detection or otherwise of glucose, sucrose, or lactose fermentation as well as gas or hydrogen sulphide production. The ability of each test coliform bacterium to utilize indole, citrate, and urea was determined using other biochemical assays. Gram staining was performed to confirm the morphology of various bacterial isolates. Standard laboratory procedures were followed for all the tests conducted [24, 26].

### 2.7. Antibiotic Susceptibility Testing

The antibiotic susceptibility testing of all identified bacterial isolates was done using the disk diffusion method. All bacterial isolates were subjected to the action of 8 antibiotic discs comprising ampicillin (10 *μ*g), gentamicin (10 *μ*g), tetracycline (30 *μ*g), cefuroxime (30 *μ*g), co-trimoxazole (25 *μ*g), chloramphenicol (30 *μ*g), cefotaxime (30 *μ*g), and ceftriaxone (30 *μ*g). The guidelines provided by the Clinical Laboratory Standard Institute (CLSI) were strictly adhered to in carrying out the test [28]. Using sterile normal saline, the bacterial suspension of each bacterial isolate was prepared and standardised to the 0.5 McFarland turbidity standard. With the help of a sterile cotton swab, the whole surface of the sterile Mueller Hinton agar plate was inoculated with the suspension of each bacterial isolate to obtain a bacterial lawn. After inoculation, the plates were allowed to dry for about 10 minutes and antibiotic discs were gently deposited on their surfaces. Incubation of the agar plates was done at 35°C for 16–24 hours.

Thereafter, measurements of the diameters of the zones of inhibition were taken using a ruler and documented. Measurements obtained were translated according to the CLSI guidelines [28]. Consequently, all bacterial isolates were first categorized as sensitive, intermediate, or resistant to each antibiotic while isolates originally classified as intermediate were retested and, eventually, categorized as sensitive or resistant. The number of antibiotics to which a bacterial isolate is resistant was divided over the total number of antibiotics tested in order to determine the multiple antibiotic resistance (MAR) index of that particular isolate [29]. A calculated value higher than 0.2 indicated that the bacterial isolate was from a potentially dangerous source where antibiotics are recurrently used [30].

As a quality control measure, *Escherichia coli* ATCC 25922 and *Klebsiella pneumoniae* ATCC 700603 standard-typed strains were assessed alongside test bacterial isolate.

### 2.8. Statistical Analysis

All data were recorded into Microsoft Excel and transferred to GraphPad Prism Software Version 6. The Chi-square test and independent *t*-test were employed to assess differences in coliform loads of fish and water samples collected from all six fish farms while descriptive statistics were performed on the data generated from the questionnaire administration and the antibiotic resistance patterns of coliform bacteria. A probability value *p* < 0.05 was considered statistically significant.

## 3. Results

### 3.1. Characteristics of Samples and Farming Practices Adopted at Fish Farms

Besides water samples, tilapia (*Oreochromis niloticus*) and/or catfish (*Clarias gariepinus*) were the main types of fish from six farms located in the Central Region of Ghana. Water was obtained from all farms, and catfish was the most commonly cultured fish species of farms sampled. Only two of the farms (1 and 5) were found to be rearing both species of fish. Either catfish or tilapia was obtained from the other four farms (Table 1).

The water used to rear fish originated from boreholes, tap water, or nearby streams. All six farms were found to use fish feed produced locally from raw materials such as rice bran, wheat bran, soybean, and groundnut in combination with agricultural waste products which were periodically supplemented with commercially formulated feed purchased from shops. It was also recorded through the interview guides that all six farms did not use antibiotics, except for instances of disease outbreaks when antibiotics and other antimicrobials are applied. It is worth noting that organic manure from poultry dropping, cow, or pig dung was used to fertilize earthen ponds (data not shown).

### 3.2. Total Coliform Loads of Water and Fish Sampled from Fish Farms

The fish-holding systems in six fish farms from which water and fish samples were obtained in this study comprised one earthen pond, four concrete tanks, and one plastic tank (Table 1). Generally, the coliform loads of the fish were lower than those of water samples (*p* < 0.05). Farm 5 recorded relatively low total coliform loads across fish and water samples. Concerning water samples, the highest total coliform load of (294.000 ± 21.000)  ×  10^4^ CFU/mL was recorded for farm 1 (*p* < 0.05). Farms 2 and 4 recorded a load of (53.900 ± 8.700 and 17.200 ± 2.000)  ×  10^4^ CFU/mL, respectively, while farm 5 recorded the least coliform load of (3.000 ± 0.700) × 10^4^ CFU/mL.

Catfish and tilapia obtained from farm 1 markedly recorded the highest total coliform load of (0.900 ± 0.030 and 0.720 ± 0.130) × 10^4^ CFU/g, respectively, followed by catfish obtained from farm 2 (0.161 ± 0.041 × 10^4^ CFU/g) and tilapia obtained from farm 5 (0.140 ± 0.02 × 10^4^ CFU/g) (*p* < 0.05).

The least total coliform load for tilapia was obtained from farm 3 while for catfish, farm 4 recorded the lowest total coliform load (*p* < 0.05) (Table 1).

### 3.3. Distribution of Coliform Bacteria according to Fish Farms and Their Antibiotic Resistance Profile

Following the performance of various biochemical assays and Gram staining, a total of 63 coliform bacteria belonging to various genera of the Enterobacteriaceae family were identified. The most predominant bacterial species across all six farms were found to be *Citrobacter freundii* (18) followed by *Klebsiella pneumoniae* (12) and *Escherichia coli* (11) (Table 2). The least represented were *Serratia marcescens* (1), *Proteus mirabilis* (1), and *Citrobacter diversus* (1). Also, the antibiotic resistance profile of the coliform bacteria is displayed in Table 3. All but one coliform bacterium (*Shigella sonnei*) was found to be resistant to ampicillin (98.4%). The next antibiotic to which most coliform bacteria showed resistance was cefuroxime (88.9%). Tetracycline, cefotaxime, and co-trimoxazole recorded 66.7%, 52.4% and 56.0% percentage resistance, respectively. Only 6.4% of the 63 bacterial isolates were resistant to gentamicin.

### 3.4. Antibiotic-Resistant Patterns Recorded in This Study and Multiple Antibiotic Resistance (MAR) Indexes

Only one coliform bacterium (*Shigella sonnei*) that was isolated from farm 1 showed no resistance to any of the 8 antibiotics used in this study. Furthermore, 18 distinct antibiotic resistance patterns were recorded as shown in Table 3. Three bacterial isolates (*Citrobacter freundii*, *Escherichia coli,* and *Klebsiella oxytoca*) were found to be resistant to all 8 antibiotics (MAR = 1.00) while 13 other bacterial isolates were resistant to 7 antibiotics (MAR = 0.88). Except for farms 1 and 3 from which 3 and 2 coliform bacteria, respectively, recorded MAR indices <0.20, all coliform bacteria recovered from farms 2, 4, 5, and 6 recorded MAR indices with ranges such as 0.25–0.88, 0.38–0.63, 0.25–0.88, and 0.25–1.00, respectively (Table 4). The remaining 58 representing 92.0% of all bacterial isolates recorded MAR index that was higher than 0.20.

## 4. Discussion

The main sources of water for fish-holding systems of most small-to-medium scale aquaculture farms in Ghana are streams, rivers, and underground water [3]. In this study, the water used to rear fish originated from boreholes, tap water, or nearby streams. Naturally, freshwater and lakes have a complex microbiota which comprises authentic aquatic species alongside other species introduced from soil, animal, and plant sources [31]. In Ghana, the majority of aquaculture ponds are semi-intensive with many farmers applying organic manure such as chicken droppings and pig and cow dung while very few apply artificial inorganic fertilizers such as hydrated lime [32]. It was reported that some farmers in Ghana applied both inorganic fertilizers in the form of hydrated lime and organic fertilizers, and these were applied not during pond preparation, but the culture period [33]. Organic manure, however, comes along with the big challenge of microbial contamination. In this study, fish and water samples obtained from six farms were found contaminated with a varying load of coliform bacteria. Coliform bacteria are rod-shaped Gram-negative bacteria non-spore-forming and motile or nonmotile bacteria that can ferment lactose to produce acid and gas when cultured at 35–37°C [34]. They are present in the environment and the faeces of all warm-blooded animals and humans. As a result, their detection may suggest faecal contaminations of food and water. It is worth noting that the coliform levels of fish ponds sampled in this study were far above the recommended limits of ≤100 *E. coli* and <10 coliforms per millilitre for wastewater suitable for use in aquaculture [35, 36].

Farm 1 recorded the highest total coliform load of (294.000 ± 21.000)   ×  10^4^ CFU/mL, (0.900 ± 0.030) ×  10^4^ CFU/g, and (0.720 ± 0.130)   ×  10^4^ CFU/g for water, catfish, and tilapia, respectively. It is also important to note that farm 1 was an earthen pond with a coliform load of water samples far above WHO tolerable limits for wastewater used in aquaculture. Water samples obtained from the concrete and plastic tanks harboured relatively lower levels of total coliforms (53.900 ± 8.700 to 3.000 ± 0.700)   ×  10^4^ CFU/mL. Nevertheless, the least coliform load of (3.000 ± 0.700) ×  10^4^ CFU/mL recorded for farm 5 was also higher than the recommended WHO limits. While *E. coli* should not be present in wholesome fresh fish, the acceptable limit for total coliform is 100 CFU per gram of fresh fish [37]. In this study, however, the mean total coliform levels of the fish samples across all fish farms ranged from (0.900 ± 0.030) × 10^4^ CFU/g to (0.012 ± 0.003)   ×  10^4^ CFU/g.

These observations point to the poor quality of the water used to raise the fish which could partly be attributed to the feeding regime adopted in the farms. Contamination may also arise from the sanitation practices at the farms as well as the source of the water as suggested in other studies [16, 38]. Under normal circumstances, fishes acquire exogenous microorganisms on their skins, gills, and gastrointestinal tracts. However, bacteria may penetrate fish muscles when fishes are raised under stressful conditions. The invasion of fish muscle by bacterial pathogens is possible when the fish are bred in ponds that contain faecal coliform levels above 10^4^ per 100 mL with the probability of the muscle penetration increasing as the contact period of the fish with polluted water is prolonged [35]. Though not performed in this study, it is plausible that some level of contamination could be detected in the fish muscle, considering the relatively high level of total coliforms per mL of the water samples.

In this study, 63 coliform bacteria were identified, with the predominant species recorded as *Citrobacter freundii* (18, i.e., 28.6%), *Klebsiella pneumoniae* (12, i.e., and 19.0%), and *Escherichia coli* (11, i.e., 17.5%). *E. coli* is a good indicator of faecal contamination, and its presence in food and water has serious life-threatening health implications to both consumers and workers at the fish farms. More stringent measures are critically needed in fish farms that will prioritize adherence to best hygienic practices as this will go a long way to help produce healthy and wholesome fishes as well as boosting productivity. The detection of coliforms in water and fish samples is a worrisome observation deserving some pragmatic measures that would enhance the productivity of cultured fish in the Central Region of Ghana in particular and in Ghana as a whole. In addition to microorganisms, it has been reported that water from aquaculture farms is a source of antibiotics and resistant bacteria [39]. It has also been observed especially in countries that engage in large-scale agriculture that most classes of antibiotics are used in animal husbandry and aquaculture without restrictions [40, 41].

Even though the managers of the farms sampled in this study stated that antibiotics were scarcely used, it was observed that 63 bacterial isolates showed relatively high levels of resistance to eight antibiotics. In Ghana, infections caused by Gram-negative bacteria are commonly treated using these antibiotics [42]. The relatively high level of resistance of bacteria to various antibiotics observed in this study connotes the existence of some antibiotic residues in fish ponds in Ghana as reported by [7]. Antibiotics may have been directly or indirectly introduced through feeds and other extraneous waste materials from homes, hospitals, and industries into water bodies that supply the fish farms. The handling and subsequent consumption of the fish carrying multidrug-resistant bacteria represent a public health threat as these bacteria may cause various human diseases that might be difficult to treat. Another probable likelihood is that antibiotic-resistant genes harboured by resistant bacteria could be passed on to other bacteria in the gastrointestinal tract of consumers and this will result in possible life-threatening infectious diseases.

The MAR indexing is recognized as an efficient and cost-effective method for bacteria source tracking [43]. As a result, the MAR index is a useful marker to ascertain the danger of pollution that could be life-threatening [44]. Generally, the majority of the coliform bacteria identified in all six farms recorded MAR indices greater than 0.2. However, a single *Shigella sonnei* isolated from farm 1 was not resistant to any of the eight antibiotics used while two bacterial isolates each recovered from farms 1 and 3 recorded MAR indices 0.13. These bacteria exhibited 18 antibiotic resistance patterns with the majority of fish farms (4 out of 6) found to harbour coliform bacteria with MAR indices greater than 0.20. Also, 92.0% of the bacterial species isolated across all farms recorded MAR indices above the 0.20 threshold. This observation suggests that these bacteria originated from potentially dangerous sources where antibiotics are regularly used [44] and were possibly introduced through faecal contamination of human or animal origin.

Based on the target in the bacterial cell, antibiotics can be divided into three groups: inhibitors of cell wall synthesis (ampicillin, cefuroxime, ceftriaxone, and cefotaxime), inhibitors of protein synthesis (gentamicin, tetracycline, and chloramphenicol), and inhibitors of metabolite synthesis (co-trimoxazole) [26]. As reported in other studies conducted in Ghana, the highest level of resistance was recorded for ampicillin in this study [16, 42, 45]. Ampicillin is a broad-spectrum penicillin that is active against many bacteria and has been used for a relatively long period. It is cheap and can easily be accessed without prescription from various drug stores in a developing country like Ghana [42]. Consequently, the resistance of most bacteria to this drug has emerged and this has been attributed to the production of *β*-lactamases. The latter are enzymes secreted by bacteria that are capable of degrading the drug. This could explain why most bacteria in this study (98.4%) were resistant to ampicillin. There are at least four generations of cephalosporins with the first synthetized commonly referred to as first-generation cephalosporins. Cefuroxime, cefotaxime, and ceftriaxone in the present study recorded an overall percentage resistance of 88.9%, 52.4%, and 33.3%, respectively. Cefuroxime is a second-generation cephalosporin while cefotaxime and ceftriaxone are third-generation cephalosporins [46] suggesting that cefuroxime has been used for a relatively longer period than the other two. Similar resistance percentages were reported in Ghana and elsewhere [16, 42, 47–50].

Contrary to ampicillin, most bacterial isolates in this study were susceptible to gentamycin resulting in the lowest resistance percentage of 6.4% as was reported in a study conducted in the Ashanti region of Ghana [17].

Just like ampicillin, chloramphenicol and tetracyclines are easily accessible over-the-counter antibiotics and have been the mainstay of antimicrobial treatment in Africa for decades. The present study, however, reports a relatively lower percentage of bacteria resistant to chloramphenicol (36.5%) than related studies in other areas of the country [42, 48].

Co-trimoxazole, also known as trimethoprim-sulfamethoxazole, is an antibiotic resulting from the synergistic combination of the two antibiotics sulfamethoxazole and trimethoprim [46]. In this study, 46.0% of all 63 coliform bacteria were resistant to co-trimoxazole. Other studies in Ghana, however, reported slightly higher percentages of bacteria resistant to co-trimoxazole than the current study [16, 42, 49].

In line with the “One Health” concept instituted by WHO in 2017, to articulate the fact that animal and human health are interrelated and connected to the well-being of the environments in which they coexist, various member-states including Ghana were tasked to develop and implement an antibiotic resistance action plan [51]. Consequently, a 5-year National Action Plan (NAP) on antimicrobial resistance (2017–2022) was officially launched in Ghana in April 2018 with two of five strategic objectives relating to the regular surveillance of antimicrobial resistance and the optimization of the administration of antimicrobial drugs in human medicine, plant production, and animal health including aquaculture.

As per timelines provided in the NAP on antimicrobial resistance, Ghana is currently at the implementation stage of these objectives. However, considering the relatively high level of resistance observed in this study, pragmatic measures must be urgently harnessed as most of these antibiotics are used in the treatment of life-threatening infections, such as tuberculosis, meningitis, diarrhoea, and respiratory infections. Furthermore, awareness of the threat of antibiotic resistance must be imparted in the community irrespective of gender, age, or level of education to impede the spreading of resistant bacteria. The public must be sensitized and educated on the importance of antibiotics and the dangers associated with antibiotic resistance. In addition to human health, animal health and environment surveillance programs must be enforced to track the development and dissemination of antibiotic-resistant bacteria. Additional preventive measures to the use of antibiotics including vaccines, probiotics, and antimicrobials from plants and other natural sources should be also considered.

## 5. Conclusions

This study has demonstrated the presence of high levels of coliform contamination of fish farms and their products in selected locations of the Central Region of Ghana. The fish feeds used in all farms sampled were locally produced from raw materials, including agricultural waste products which are sometimes supplemented with commercially formulated feed. Water samples across all farms recorded significantly higher coliform loads than tilapia and catfish samples. Although farmers stated that antibiotics were occasionally used alongside other antimicrobials, especially during disease outbreaks, coliform bacteria isolated exhibited various antibiotic resistance patterns. Four out of six fish farms harboured some coliform bacteria with MAR indices above 0.2, an indication that these bacteria originated from potentially dangerous sources where antibiotics are regularly used. It further suggests that the bacteria were possibly introduced through faecal contamination of human or animal origin. Stringent procedures as advocated in the Nation Action Plan on antimicrobial resistance must earnestly be implemented by regulatory bodies such as the Food and Drugs Authority and the Fisheries Commission to regulate activities of managers of aquaculture farms. These regulatory bodies ought to periodically train consumers, vendors, and fish farm managers on food safety and also regulate the activities of the latter. Extensive research that will cover other regions of the country is required to obtain a clear understanding of ways to effectively and sustainably tackled antibiotic resistance in fish farms.

## Figures and Tables

**Table 1:** Total coliform loads of fish and water samples from various fish farms.

Farm	Type of fish-holding facility	Mean total coliform load of water ( × 10^4^ CFU/mL) and fish ( × 10^4^ CFU/g) samples		
		Water	Fish	
			Catfish	*Tilapia*
1	Earthen pond	294.000 ± 21.000^a^	0.900 ± 0.030	0.720 ± 0.130
2	Concrete tank	53.900 ± 8.700^b^	0.161 ± 0.040	_^*∗*^
3	Concrete tank	14.800 ± 4.200	_^*∗*^	0.012 ± 0.003^e^
4	Concrete tank	17.200 ± 2.000^c^	0.013 ± 0.004^f^	_^*∗*^
5	Concrete tank	3.000 ± 0.700	0.120 ± 0.010	0.140 ± 0.02
6	Plastic tank	4.000 ± 0.500^d^	0.130 ± 0.006	_^*∗*^

_^*∗*^Type of fish not sampled due to nonavailability at the farm. “a” depicts the significant difference between the mean total coliform load of water and catfish in farm 1. “b” depicts the significant difference between the mean total coliform load of water and catfish in farm 2. “c” depicts the significant difference between the mean total coliform load of water and catfish in farm 4. “d” depicts the significant difference between the mean total coliform load of water and catfish in farm 6. “e” depicts the significant difference between the mean total coliform load of tilapia in farm 3 and those from other farms. “f” depicts the significant difference between the mean total coliform load of catfish in farm 4 and those from other farms.

**Table 2:** Distribution of 63 bacterial isolates and their antibiogram (percentage in brackets).

Organism	Number of isolates	Frequency of bacterial isolates resistant to antibiotics listed below							
		COT	GEN	CRX	CHL	CTR	CTX	AMP	TET
*Serratia marcescens*	1	1 (100.0)	0 (0.0)	1 (100.0)	1 (100.0)	1 (100.0)	1 (100.0)	1 (100.0)	1 (100.0)
*Citrobacter freundii*	18	4 (22.2)	1 (5.6)	17 (94.4)	3 (16.7)	5 (27.8)	10 (55.5)	18 (100.0)	12 (66.7)
*Klebsiella oxytoca*	5	5 (100.0)	1 (20.0)	5 (100.0)	5 (100.0)	4 (80.0)	4 (80.0)	5 (100.0)	5 (100.0)
*Escherichia coli*	11	6 (54.5)	1 (9.1)	9 (81.8)	4 (36.4)	5 (45.5)	6 (54.5)	11 (100.0)	8 (72.8)
*Enterobacter aerogenes*	2	1 (50.0)	1 (50.0)	1 (50.0)	1 (50.0)	1 (50.0)	1 (50.0)	2 (100.0)	2 (100.0)
*Klebsiella pneumoniae*	12	9 (75.0)	0 (0.0)	12 (100.0)	6 (50.0)	4 (33.3)	5 (41.7)	12 (100.0)	11 (91.7)
*Edwardsiella tarda*	3	0 (0.0)	0 (0.0)	3 (100.0)	0 (0.0)	1 (33.3)	3 (100.0)	3 (100.0)	1 (33.3)
*Salmonella typhi*	5	1 (20.0)	0 (0.0)	4 (80.0)	1 (20.0)	0 (0.0)	2 (40.0)	5 (100.0)	0 (0.0)
*Proteus mirabilis*	1	0 (0.0)	0 (0.0)	1 (100.0)	0 (0.0)	0 (0.0)	0 (0.0)	1 (100.0)	0 (0.0)
*Citrobacter amalonaticus*	2	2 (100.0)	0 (0.0)	2 (100.0)	1 (50.0)	0 (0.0)	0 (0.0)	2 (100.0)	1 (50.0)
*Shigella sonnei*	2	0 (0.0)	0 (0.0)	0 (0.0)	0 (0.0)	0 (0.0)	0 (0.0)	1 (50.0)	0 (0.0)
*Citrobacter diversus*	1	0 (0.0)	0 (0.0)	1 (100.0)	0 (0.0)	0 (0.0)	1 (100.0)	1 (100.0)	1 (100.0)
Total	63	29 (46.0)	4 (6.4)	56 (88.9)	22 (34.9)	21 (33.3)	33 (52.4)	62 (98.4)	42 (66.7)

COT = co-trimoxazole; GEN = gentamicin; CRX = cefuroxime; CHL = chloramphenicol; CTR = ceftriaxone; CTX = cefotaxime; AMP = ampicillin; TET = tetracycline.

**Table 3:** Antibiotic resistance patterns observed among 63 coliform bacteria.

Number of antibiotics	Resistance pattern	Number of bacterial isolates
0	No resistance	1
1	AMP	4
2	CRX + AMP	8
2	AMP + TET	1
3	CRX + CTX + AMP	5
3	COT + AMP + TET	1
3	COT + CRX + AMP	1
3	CRX + AMP + TET	8
3	CRX + CTR + CTX	1
4	COT + CRX + AMP + TET	3
4	CRX + CTR + CTX + AMP	1
4	CRX + CTX + AMP + TET	4
5	COT + CRX + CTR + CTX + AMP	1
5	COT + CRX + CHL + CTX + AMP	1
5	COT + CRX + CHL + AMP + TET	3
6	COT + CRX + CHL + CTX + AMP + TET	2
6	COT + CRX + CTR + CTX + AMP + TET	2
7	COT + CRX + CHL + CTR + CTX + AMP + TET	13
8	COT + GEN + CRX + CHL + CTR + CTX + AMP + TET	3
Total		63

COT = co-trimoxazole; GEN = gentamicin; CRX = cefuroxime; CHL = chloramphenicol; CTR = ceftriaxone; CTX = cefotaxime; AMP = ampicillin;TET =  tetracycline.

**Table 4:** Antibiotic resistance profile and MAR of coliforms from fish ponds.

Source	Sample type	Isolate code	Isolate ID	Antibiotic resistance pattern	MAR index
Farm 1	Water	A1	*Edwardsiella tarda*	CRX CTX AMP	0.38
		A2	*Escherichia coli*	AMP	0.13
		A3	*Shigella sonnei*	AMP	0.13
		A5	*Escherichia coli*	COT CRX CTR CTX AMP TET	0.75
		A6	*Shigella sonnei*	No resistance	0.00
	Catfish	H_1_1	*Salmonella typhi*	CRX CTX AMP	0.38
		H_1_3	*Citrobacter freundii*	CRX CTX AMP	0.38
		H_1_2	*Edwardsiella tarda*	CRX CTX AMP TET	0.50
	*Tilapia*	H_2_2	*Citrobacter freundii*	CRX CTX AMP	0.38
		H_2_3	*Citrobacter freundii*	CRX CTX AMP TET	0.50
		H_2_1	*Citrobacter diversus*	CRX CTX AMP	0.38

Farm 2	Water	C3	*Klebsiella oxytoca*	COT CRX CHL CTR CTX AMP TET	0.88
		C1	*Citrobacter freundii*	CRX AMP	0.25
		C2	*Citrobacter freundii*	CRX AMP	0.25
	Catfish	F3	*Citrobacter amalonaticus*	COT CRX CHL AMP TET	0.63
		F2	*Salmonella typhi*	COT CRX CHL CTX AMP	0.63
		F1	*Proteus mirabilis*	CRX AMP	0.25

Farm 3	Water	B3	*Escherichia coli*	AMP	0.13
		B4	*Citrobacter freundii*	CRX AMP TET	0.38
		B1	*Salmonella typhi*	AMP	0.13
		B2	*Edwardsiella tarda*	CRX CTR CTX AMP	0.50
	*Tilapia*	G3	*Citrobacter freundii*	CRX AMP	0.25
		G1	*Salmonella typhi*	CRX AMP	0.25
		G2	*Salmonella typhi*	CRX AMP	0.25

Farm 4	Water	D2	*Klebsiella oxytoca*	COT CRX CHL AMP TET	0.63
		D1	*Klebsiella pneumoniae*	COT CRX AMP TET	0.50
		D3	*Klebsiella pneumoniae*	COT CRX CHL AMP TET	0.63
		E2	*Citrobacter amalonaticus*	COT CRX AMP	0.38
	Catfish	E1	*Klebsiella pneumoniae*	COT CRX AMP TET	0.50
		E3	*Citrobacter freundii*	COT AMP TET	0.38

Farm 5	Water	K1	*Enterobacter aerogenes*	COT CRX CHL CTR CTX AMP TET	0.88
		K2	*Klebsiella pneumoniae*	CRX AMP TET	0.38
		K5	*Klebsiella pneumoniae*	COT CRX CHL CTR CTX AMP TET	0.88
		K4	*Klebsiella pneumoniae*	COT CRX CTR CTX AMP TET	0.75
		K6	*Klebsiella pneumoniae*	COT CRX CHL CTR CTX AMP TET	0.88
		K7	*Klebsiella pneumoniae*	COT CRX CHL CTX AMP TET	0.75
		K3	*Enterobacter aerogenes*	AMP TET	0.25
	Catfish	I1	*Escherichia coli*	COT CRX CHL CTR CTX AMP TET	0.88
		I6	*Citrobacter freundii*	CRX CTX AMP TET	0.50
		I3	*Citrobacter freundii*	CRX AMP	0.25
		I4	*Citrobacter freundii*	CRX AMP TET	0.38
		I5	*Escherichia coli*	CRX AMP TET	0.38
		I7	*Citrobacter freundii*	CRX AMP TET	0.38
		I2	*Citrobacter freundii*	CRX CTX AMP TET	0.50

Farm 6	Water	L5	*Klebsiella pneumoniae*	COT CRX AMP TET	0.50
		L1	*Escherichia coli*	COT CRX CHL CTR CTX AMP TET	0.88
		L2	*Escherichia coli*	CRX AMP TET	0.38
		L3	*Escherichia coli*	CRX AMP TET	0.38
		L4	*Escherichia coli*	COT CRX CTR CTX AMP	0.63
	*Tilapia*	*J*_1_4	*Citrobacter freundii*	COT CRX CHL CTR CTX AMP TET	0.88
		*J*_1_5	*Klebsiella pneumoniae*	COT CRX CHL CTR CTX AMP TET	0.88
		*J*_1_6	*Klebsiella pneumoniae*	CRX AMP	0.25
		*J*_1_7	*Citrobacter freundii*	COT CRX CHL CTR CTX AMP TET	0.88
		*J*_1_3	*Citrobacter freundii*	COT GEN CRX CHL CTR CTX AMP TET	1.00
		*J*_1_1	*Serratia marcescens*	COT CRX CHL CTR CTX AMP TET	0.88
		*J*_1_2	*Citrobacter freundii*	CRX CTR CTX	0.38
	Catfish	*J*_2_6	*Citrobacter freundii*	COT CRX CHL CTR CTX AMP TET	0.88
		*J*_2_3	*Klebsiella oxytoca*	COT CRX CHL CTR CTX AMP TET	0.88
		*J*_2_4	*Escherichia coli*	COT GEN CRX CHL CTR CTX AMP TET	1.00
		*J*_2_5	*Klebsiella oxytoca*	COT CRX CHL CTR CTX AMP TET	0.88
		*J*_2_7	*Klebsiella pneumoniae*	CRX AMP TET	0.38
		*J*_2_1	*Klebsiella oxytoca*	COT GEN CRX CHL CTR CTX AMP TET	1.00
		*J*_2_2	*Escherichia coli*	COT CRX CHL CTX AMP TET	0.75

## Data Availability

The datasets obtained during this research are available from the corresponding author upon request.
